# Fournier's Gangrene and the Reconstructive Challenges for the Plastic Surgeon

**Published:** 2016-08-29

**Authors:** David Izadi, James Coelho, Sameer Gurjal, Faisal Salim

**Affiliations:** Royal Devon and Exeter Hospital NHS Foundation Trust, Exeter, United Kingdom

**Keywords:** Fournier's, gangrene, scrotum, perineum, reconstruction

**Figure:**
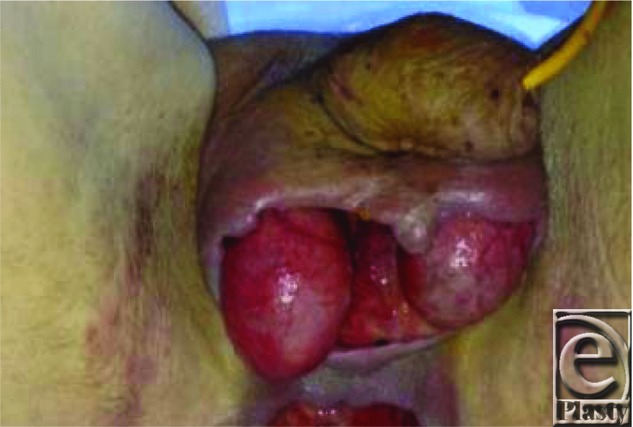


**Figure:**
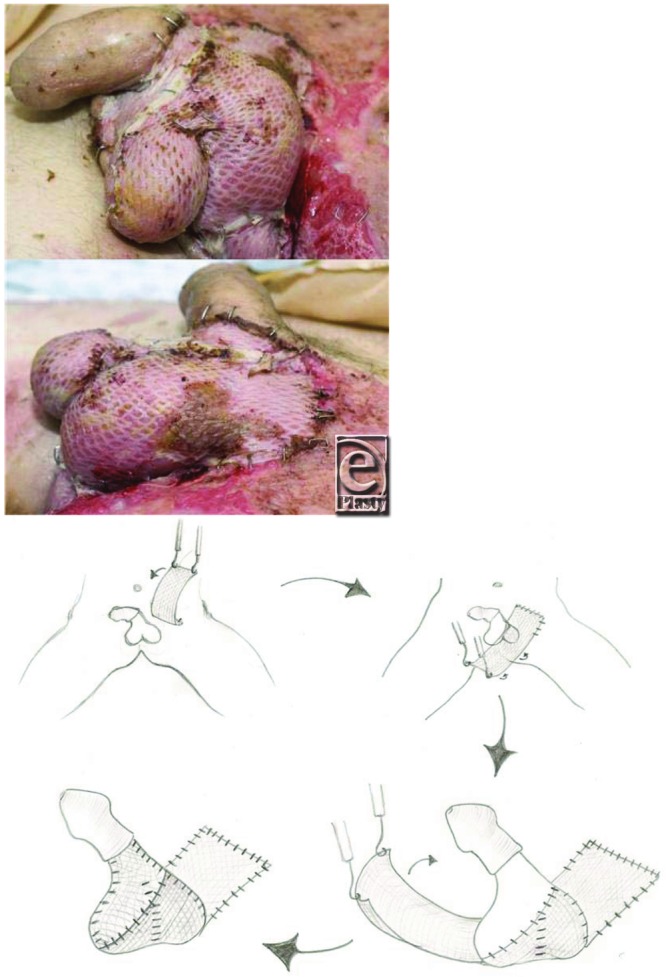


## DESCRIPTION

A 37-year-old male patient presented with a painful swelling of his scrotum for 2 days, temperature of 38.5°C, with scrotal discharge for 36 hours. He was an active intravenous drug user, injecting his groin 3 days earlier. He underwent emergency surgical debridement (Figure 1), followed by reconstruction with split-thickness skin grafts (Figure 2).

## QUESTIONS

1. **What is Fournier's gangrene?**
2. **What is the emergency management of Fournier's gangrene?**
3. **What are the reconstructive options for perineal and scrotal defects?**
4. **Describe how to use a split-thickness skin graft for a 1-stage reconstruction of a scrotal defect?**

## DISCUSSION

Fournier's gangrene is a rare necrotizing fasciitis infection, primarily of the skin and subcutaneous tissues of the genitals, perineum, and abdominal wall. It can rapidly spread to surrounding soft tissues and has a mortality rate of up to 40%. It was first described as an idiopathic gangrene of the penis and scrotum in 1883 by the French dermatologist Jean-Alfred Fournier.^1^ Fournier's gangrene is 5 to 10 times more common in men, with 60- to 80-year-olds most affected. Fournier's gangrene is associated not only with comorbidities, especially diabetes mellitus, but also with renal failure, inflammatory bowel disease, morbid obesity, alcoholics, malignancy, and HIV infection. It results from polymicrobial infections, predominantly anaerobic bacteria from a colorectal, genitourinary, or cutaneous infection of the genitals, perineum, or anorectal regions. The most common pathogens are streptococcal species, staphylococcal species and *Escherichia coli*, and less frequently fungal species.^2^ It is likely that within a polymicrobial environment, the secretion of various enzymes, including collagenases and lecithinase, is responsible for the breakdown of fascial planes, tissue necrosis, and the rapid spread of the infection. The diagnosis of Fournier's gangrene is often made clinically, where patients present with pyrexia, spreading erythema, pain, swelling, crepitus, and tissue necrosis, and is seen in more than 60% of cases.^3^ The main tool used to predict severity of the disease and mortality risk is the Fournier's gangrene severity index (FGSI). FGSI can predict mortality with a probability of 75% and survival with a probability of 78% for patients with Fournier's gangrene.^4^

Unless aggressively treated, patients with Fournier's gangrene will become septic and go onto develop multiorgan failure, which is the main cause of their death. Principal treatment includes hemodynamic resuscitation, broad-spectrum antibiotics, and surgical debridement. Surgical debridement of infected tissues is essential until healthy viable tissues are reached, and, in addition, urinary and fecal diversions may be necessary. Multiple trips to the operation theater for reassessment, dressing changes, and further debrided of wounds are common, and the soft-tissue defects are often temporarily dressed with negative pressure wound dressings. After the patient has stabilized, the resultant soft-tissue defects will require delayed reconstruction.

Scrotal reconstruction following debridement for Fournier's gangrene is a complex problem where consideration should be given to skin cover, aesthetics, and function of the testes. Small scrotal defects can be left to granulate and heal by secondary intention. Defects less than 50% of the scrotum can be reconstructed using scrotal advancement flaps. Burying the testes subcutaneously in medial thigh pockets is not popular with the patient and has a risk of spermatic cord necrosis. Split-thickness skin grafts can be used as long as the tunica vaginalis is intact.^5^ Grafts are easy to use and have superior aesthetic results to bulkier flap reconstructions. For more extensive defects, especially where the tunica vaginalis is no longer present, local pedicled muscle flaps, namely the gracilis or vertical rectus abdominis muscle flaps,^6^^,^^7^ or local fasciocutaneous flaps^5^^,^^8^ such as the anteromedial thigh flap, anterolateral thigh flap or pudendal artery thigh flap can be used.

Meshed split-thickness skin graft can be used to drape around the exposed testes as a scrotal sling in a 1-stage reconstruction. Simply harvest 1 long 30-cm length split-skin graft using an air dermatome at 0.012-μm thickness. Mesh 1-1.5 ratio and secure graft at one edge before draping around the testes to achieve skin cover and a good aesthetic outcome (see diagram in Figure 2).

In summary, Fournier's gangrene is a life-threatening soft-tissue infection affecting the scrotum and perineum. Early surgical excision, together with broad-spectrum antibiotics, is required for the emergency management. Reconstruction of the soft tissues depends on the anatomical defects, with special consideration made for the scrotum.
